# Surgery

**Published:** 1878-01

**Authors:** 


### III. Surgery.

Treatment of the Transverse Fractures of the Patella and the Olecranon.—Max Shede. (*Reports of the City Hospital at Berlin.*) It is a well-known fact that the fragments of a transversely broken patella are so seldom united by osseous substance that sometimes the possibility of this kind of union has been positively denied. Malgaigne relates that Pibrac set up a prize of 100 Louis d'or for the man who could show him a fractured patella united by a thoroughly ossified callus. Since that time the possibility of this kind of healing has been established unquestionably.

But this result is now-a-days attained almost as seldom as at the time of Malgaigne and Pibrac.

In consideration of the following facts: *first*, that in longitudinal and comminuted fractures of the patella, osseous union always takes place without difficulty; *second*, that the periosteum of the patella possesses such a power of producing osseous substance that almost the whole of the bone can be reproduced if it were lost by necrosis after a compound fracture; *third*, that stalactite shaped masses of luxuriating callus are found at the edges of separated fragments of the patella; in view of these facts there can be no doubt but that it cannot be any internal physiological cause, but only some external unfavorable conditions, which prevent the osseous union of a transverse fracture of the patella.

Longitudinal and comminuted fractures heal with osseous tissue, because the fragments remain in close apposition. The transverse fractures are united only by fibrous or ligamentous substances, because the fragments remain more or less separated.

Not one of all the numerous, often very ingenious, apparatus and bandages, proposed for the treatment of the above mentioned fracture, has kept up its reputation. Generally the surgeon is satisfied with obtaining a ligamentous union by a layer two or three "lines" broad. Nevertheless, the author does not believe that this unsatisfactory result is caused wholly by faults of our bandages not being able to achieve a complete and permanent coaptation of the fragments. Many of these bandages would certainly give better results if they could be applied immediately after the injury had taken place. But this is generally impossible, owing to great accumulation of blood within the synovial sac, and to considerable swelling of the surrounding soft parts. It is necessary to wait for the absorption of the exudation, and the appropriate treatment of the fracture cannot commence until the most precious time for the consolidation of fractures has expired.

In this way several circumstances come together to prevent the osseous union, to-wit: the considerable extravasation of blood, which is commonly accepted as protracting the consolidation of fractures, and the retarded coaptation of the fragments. The logical consequence of the above train of ideas must lead to an early operative evacuation of the fluid from the capsular cavity, as the condition of obtaining more favorable results. As it now is a well established fact that "puncture of a joint, if performed antiseptically, is absolutely free from danger," the author thought himself entitled to make the experiment.

The author has employed this treatment in five cases of transverse fractures of the patella. The puncture of the joint always proved entirely harmless. Nevertheless, the two first cases healed by a ligamentous union, although the interposed fibrous matter was very thin, and scarcely measurable. This unsatisfactory result was caused by failures in the after-treatment, which the author afterward learned to avoid. But the three last cases healed by a perfectly solid, osseous union. The treatment was as follows: He punctured the capsular cavity, and, if needed, the bursa patellæ, too, using a strong trocar, and all antiseptic precautions.

There is no difficulty in letting out the blood, which usually is fluid and mixed with synovia. The puncture is followed by injections of a three per cent. solution of carbolic acid; they are repeated until the fluid returns perfectly clear. He then takes out the trocar and closes the wound, placing a small piece of silk protective, and a little pad af salicylated cotton upon it.

The exact coaptation of the fragments is obtained by means of long, two or three centimeters broad, strips of adhesive plaster, which, at the same time, keep the salicylated cotton in its place. The upper strips, which are to draw the upper fragments downward, must cross each other on the calf and finally reach the anterior aspect of the leg. The strips for the lower fragment must encircle the thigh in a similar way. Then the whole extremity is tightly bandaged with flannel-rollers, the turns of which must be applied like a figure 8 bandage around the knee-joint, so as to strengthen the effect of the plaster-strips. At last, a plaster-of-Paris bandage is applied from the ankles to the hip of the completely extended extremity. Furthermore, the author thinks it necessary that this bandage should be renewed, during the first two or three weeks several times, at short intervals. Especially the first change of the bandage must take place before eight days have elapsed, the decrease of the œdematous swelling having made the bandage so loose, as to necessitate its renewal. The author, after eight days, always found a considerable space between the bandage and the limb, the plaster-straps quite loose, and the fragments a little distant from each other. A new bandage, easily corrected the deficiency. But the second and third bandages will show the same condition after eight days, owing to a steady decrease of the swelling of the soft parts around the joint, and the atrophy of the leg from inactivity.

It is unnecessary to mention that the slightest flexion of the limb must be avoided while the bandage is changed. If it is shown by the second or third change of the bandage, that no further decrease of the joint in size has taken place, and that the fragments remain in close opposition, the new bandage can remain during the rest of time required for healing.

As a general rule, the duration of the treatment by plaster-of-Paris bandages, has been six weeks. The after treatment must have two objects in view: *first*, to protect the young osseous scar against violent injuries; *second*, to restore by degrees the flexibility of the joint. A false step causing the knee to be bent suddenly, would, at this period, most likely sever the fresh union.

None of the numerous varieties of elastic knee-caps or similar bandages, are suitable to this purpose. Only a solidly constructed apparatus, consisting of articulated splints, extending from the foot to the hip, will keep the patella safe, and assure the definite healing. This apparatus must be provided with a hinge opposite the knee, to permit a gradual flexion, which, commencing with 20°, is increased a little every four weeks. It is not advisable to let the patient walk without the apparatus, until four or six months have elapsed. In the meantime the flexibility of the joint must be acted upon by means of passive motions, massage, baths, douches. C. F.

THREE CASES OF ORAL ABSCESS.—T. Pauly.—(*Berliner Klinische Wochenschrift*, 1877, *N.* 22.)

I. *Idiopathic Retropharyngeal-abcess in a Baby.*—The author was called to see a baby in the state of extreme suffocation. Exploring with the finger he found on the right side of the pharynx behind the epiglottis, a fluctuating tumor of the size of a pigeon's egg. Fearful of a sudden aspiration of the pus into the lungs if he made a free incision, he preferred to make a mere puncture. The baby instantly felt relieved and respired with ease. When, in the afternoon, the contents of the abcess had increased again, he opened it thoroughly with a tenotome and evacuated the pus through the mouth over the tongue, having bent the head of the baby forward.

II. *Abcessus glosso-epiglotticus.*—A baby three months old had difficulty in sucking. Examining with the finger the doctor found an abcess between the tongue and the epiglottis. Having at first employed a trocar, the cavity was filled again, whereupon he lanced thoroughly with perfect success.

III. *Abcess in the Tongue.*—A farmer, 17 years of age, suffered from difficulty in swallowing caused by an abcess in the tongue about an inch above the ligamentum glosso-epiglotticum. The diagnosis could be made by means of the laryngoscope. He recovered after incision. C. F.

Excision of the Knee Joint, with Transverse Division of the Patella.—R. Volkmann. (*Deutsche Medicinische Wochenshrift*, 1877. N. 33.) During the last three years, the author has performed twenty-one excisions of the knee joint, and lost only one patient, a child, which died of tubercular meningitis, three weeks after the operation. He justly points to the importance of combining the total extirpation of the fungous or tubercular capsule with the excision of the diseased parts of the bones. The extirpation of the whole capsule necessitates for all joints a somewhat larger division of the soft parts. For the shoulder, elbow and hip joints, the longitudinal incision will do, but for the knee joint the author thought it necessary to devise another incision, which, while it permits the accurate removal of the capsule and the surrounding fungous tissue, would, at the same time, preserve the integrity of the quadriceps femoris. For this purpose he cut across the middle of the patella, divided the latter by the saw into equal halves, and, after having completed the excision in the usual way, he united the two halves of the patella by means of cat-gut sutures. This method has, in several cases, proved very useful. For very extensive excisions and in case of unusually great infiltration of the soft parts he recommends an H-cut. If the pieces are scrupulously coaptated by cat-gut sutures, the patella will become quite firm in fourteen days. The surfaces of the epiphyses must be united by two cat-gut sutures. If the patella is adherent to the condyles of the femur, it must be detached with the chisel.

The author relates four cases operated on by the above method. In all, the disease had reached such an extent as not to allow a favorable prospect for a good result.

In three of these excisions, the patella could be left entire, or only its cartilaginous surface had to be scraped off. In the fourth case there were three carious cavities filled with dead bone and yellow cheesy matter in the patella, of which only the external plate could be left. Nevertheless, the two thin plates healed firmly together without dislocation. The joints operated upon quickly consolidated and proved useful, in spite of the fact that considerable portions of carious bone had been removed in two cases. C. F.

Parenchymatous Injections of Acetic Acid in Carcinoma.—Th. Gies (*Deutsche Zeitschrift für Chirurgie*, bd. 8, p. 279). A man, 62 years old, had a glandular tumor under the horizontal branch of the lower jaw. The tumor was supposed to be a carcinoma following the extirpation of a small growth in the lower lip. The tumor was extirpated, and the microscope confirmed the diagnosis.

Some time afterwards, the patient felt pain in the tongue, owing to a carcinomatous ulcer in the left half of the tongue, not far from the epiglottis. The degenerated portion was excised after ligature of the arteria lingualis and division of the lower jaw.

Seven months later, two new tumors developed, a smaller one near the left border of the lower jaw, and a larger one below on the neck. The latter disappeared under treatment with ice, but the former remained stationary. A year after the tongue had been extirpated, a rapidly growing tumor developed in the neighborhood of the right submaxillary gland. The growth did not decrease in size when treated with ice, and it soon commenced to dislocate the trachea. A small piece of the tumor, taken out by the harpoon, proved to be carcinomatous by a microscopic examination.

The author then injected into the tumor, once every week, the whole contents of a Pravaz syringe filled with a solution of 1 part acid. acetic. glaciale to 3 parts of water.

Through the same puncture in the skin, he plunged the needle into different parts of the growth, emptying the syringe gradually, so as to spread the contents through the whole tumor. Warm poultices were applied afterward. Great swelling soon followed. On the 10th day, he made a deep incision with a pointed knife, and inserted a drainage tube, through which an offensive, ichorous matter was discharged. After 17 days the discharge ceased, and at the end of four months, there remained only a small, hard tumor, lodged deeply in the tissues. Soon afterward the same treatment was applied to the tumor near the margin of the lower jaw, and to a new one in the left cheek, with a similar good result. A new tumor now presented itself below the left ear; it increased to the size of a hen's egg, was very hard, and showed, on micoscopic examination, a more abundant framework of connective tissue than did the former tumors. Injections again were resorted to, but, considering the greater firmness of this growth, the author injected the whole contents of a syringe two or three times daily. During eleven days, twenty-five syringes filled with the solution of acetic acid were injected. The injections into this tumor produced great pain, the former injections in the soft growths having been almost painless. On the twelfth day the incision was made. The suppuration lasted three weeks, and the tumor disappeared almost entirely.

A similar favorable result was obtained by the author in another case. A woman, exceedingly afraid of an operation, had a soft carcinomatous tumor in the breast. During 10 days a syringe-full was injected daily, and on the 11th day the incision was made. After a few weeks the suppuration had ceased, and four weeks later only a small painless tumor remained, as big as a nut, in the depth of the breast.

In both these cases, the author made the injections for the purpose of destroying the new formation through suppuration. For that reason he used solutions of acetic acid, strong enough to produce cauterization of the tissues.

He recommends paying more attention to this method than generally has been done, because it may be a very useful one in cases not amenable to operations; if it does not cure it it may, at any rate, restrain the rapid growth of the neoplasm. The author's cases are yet of too recent a date to form a definite opinion of the ultimate fate of the patients.

Treatment of Goitre by Interstitial Injections. (*Journ. de Médecine*, *Nov.*, 1877.) In a review published in the *Annales des Maladies du Larynx et des Oreilles*, M. Cazalis studies the different modes of treating goitre, especially the method of Luton. This method is particularly applicable to parenchymatous and fibrous goitres. By this process, in carrying the iodine to the center of the tumor, Luton has often cured goitres otherwise incurable. He employs the ordinary hypodermic syringe, but it is best to have it gold or nickel-plated. The piston should fit accurately, so as to overcome the resistance of the tumor. Luton recommends the ordinary tincture of iodine, from 1 to 5 grammes, or the following: distilled water 40 grammes, tr. iodine 20 grammes, iodide of potassium 1 gramme; dose 15 to 40 drops. The solution should only penetrate the substance of the gland, and it is well to anæsthetize the part with ice or the ether spray. The veins should be avoided, and the introduction of air as well.

The symptoms vary according to the strength and amount of the liquid injected. Small doses produce only an uneasiness or slight smarting in the region of the neck. With stronger injections, more or less painful radiations are felt towards the jaw and ears. Sometimes acute iodism results, the pulse and the temperature rise, the neck swells, but the inflammatory accidents subside generally in two or three days, and there is felt in the interior of the gland a hard kernel which diminishes little by little with the gland. In order to render the pain and perhaps the irritation less, Heller adds 15 milligrammes of morphine to the injection. Suppuration is rare.

Although this method usually gives more speedy results than medical treatment, it often requires several months before definite results are obtained. It is often necessary to renew the injections. Morell Mackenzie, who has had a large experience with this method, renews the injections about every ten days. In this case it is best to make them at different points. If the goitre presents several lobes, they may be taken successively, although the injection of one may suffice. If the goitre is very large, two injections, some distance apart, may be made at the same sitting.

The vascular goitre should not be treated by this method. For the fibrous goitre it is especially applicable, and Mackenzie has had 59 cases in 73 cases treated by this method. L. W. C.

Diphtheria and Tracheotomy.—Kroenlin. [*Archiv für Klin. Chirurgie*, 1877, bd. xxi., Sept. 2.) In the first portion of the communication the author gives a statistical report of 567 cases of diphtheria, which came under the care of Professor von Langenbeck, from January 1st, 1870, to July 31st, 1876. Of the 567 cases, 539 were brought to the hospital with the malady, while 28 contracted it there, while under treatment for other diseases.

Of the 567 patients, 379 (or 66.4 per cent.) died, while 190 (or 33.6 per cent.) recovered. Another table shows that the number of cases of diphtheria has steadily increased with each succeeding year, while the rate of mortality has constantly decreased. In 1869, the mortality in 72 cases of this affection was 86.0 per cent. The reason for this fact is not understood.

Only eight of those cases occurred in patients between 18 and 41 years; the largest number (102 or 18.3 per cent.) being found among children three or four years of age. It may be considered, as a general rule, that the younger the patient the more unfavorable is the prognosis, and that tracheotomy has no influence to alter this fact.

Tracheotomy was performed in all cases of laryngeal stenosis, regardless of pulmonary complications. The operation was performed 504 times; of these, 357 (70.8 per cent.) died; 85 of those operated upon were infants under two years, 11 of this class recovering, the youngest being seven months of age. In cases of surgical interference, death generally occurred on the first or second day, while after the fifth day fatal terminations were seldom recorded.

The occurrence of 28 cases of diphtheria in patients under treatment for other surgical diseases at the clinique (having a mortality of 18, or 64.2 per cent.) demonstrated that the collection of large numbers of diphtheritic patients in the wards of a hospital during many years, has an injurious effect upon the salubrity of the institution.

In the second part of his work, Dr. K. attempts to settle other questions upon the observation of 241 cases of which he possessed complete and minute records. In 46 cases there were false membranes upon, and ulceration of, the nasal fossæ, but not of the pharynx; all of these were subjected to operation because of great tracheal stenosis. Of the remaining 195 cases, 31 exhibited no impediment to respiration, and consequently were not operated upon. The mortality among those operated upon was 71.7 and 73.7 per cent. respectively, while of those not tracheotomised, but 32.2 per cent. resulted fatally — a fact which proves that the local manifestation of the disease in the respiratory tract constitutes a fearful complication.

In those cases where the respiration was not perfectly free after the operation, the mortality was 25.2 per cent. greater than where it was; the course of those cases, where large masses of croupous membrane were expelled, was very unfavorable. Twenty-two of the children were brought to the clinique in a state of advanced asphyxia; they were operated upon without anæsthetics; two recovered.

In 66 cases the canula was removed between the third and seventeenth day; in one case (owing to granulations) not until six months had expired; of these 67 cases, 16 (23.8 per cent.) snbsequently died (causes: collapse 1, diphtheritic nephritis 1, pneumonia 2, exhaustion, due partially to difficult deglutition, 12).

Obstruction in deglutition was observed in 42 cases. It occurred between the first and forty-fourth day, but had no relation to the operation; the mortality of this class was 61.3 per cent.

Diphtheritic exudation on the wounds after tracheotomy was recorded in 50 cases, of which 28 died; beside this an exanthema was occasionally noticed in the thorax (anterior and posterior), neck, and in the immediate locality of the incision; albuminuria also was frequently observed.

Tracheotomy (except in asphyxiated cases) was performed while the patients were thoroughly anæsthetised with chloroform.

Treatment: After various medicaments had proved unsatisfactory, the treatment adopted since May, 1864, has been the local application of chlorine water penciled upon the affected portions of the pharynx every hour or two; a few drops of a diluted mixture (aq. chlorini 1 to aq. pura 3 parts) were introduced into the canula. Diphtheritic abscesses and suppuration of the submaxillary lymphatic glands occurred 5 times in 241 cases, and in one case suppurative mediastinitis.

A microscopic examination of the pus showed micrococci in abundance.

Intussusception Cured by Reduction.—Prof. Schillbach at Jena. (*Centralblatt für Chirurgie*, No. 37, 1877.) On the 31st of July, Professor Schillbach was called to see a boy, 13 years old, who, having a few days previously suffered from a slight diarrhœa, was suddenly seized with intense pain in the abdomen, and with vomiting. Those symptoms had lasted 24 hours in spite of all applied remedies. Besides great depression of the vital powers, Professor Shillbach now found some tension of the whole abdomen, but more marked on the left side and upward from the umbilicus. Here about two inches above and to the left of the umbilicus, he detected an oblong curved body one and a half inches in diameter and four to five inches in length, the boundary of the upper portion of which was well marked, but the lower margin, indefinite; on percussion this body gave a subdued resonance, the surrounding parts being decidedly tympanitic. A slight pressure on the tumor produced great pain, while the remaining portions of the abdomen were not sensitive; the inguinal regions showed slight resonance and some tenderness; at no portion of the abdomen were the bowels abnormally distended by gases. No evacuation of the bowels had occurred after the severe symptoms transpired; the vomiting had ceased since 24 hours; the pulse was 100 and of medium fullness; the temperature of the skin was not unusually high, the hands somewhat cool; slight thirst and no appetite.

As there were no alarming symptoms, for the night Professor Schillbach directed cold compresses over the abdomen, postponing until the next visit the diagnosis and appropriate treatment.

The next day (August 1st) the nausea returned, the symptoms otherwise being those of the previous day; exploration per rectum furnished only negative results, the finger on withdrawing showing traces of excrements, an enema returned bloody water but no fæces.

The symptoms thus excluding the presumption of hernia, volvulus, diffuse peritonitis or compression by tumors, there remained but one diagnosis, viz., that of intussusception, which explained the discharge of bloody water, due to contusion of the mucous membrane in the process of invagination.

As the case was recent, with no evidence of diffuse peritonitis, and sufficient time had not elapsed for plastic adhesions, the Doctor was persuaded to try reduction of the intussusception. For this purpose he employed a flexible rubber tube, with apertures at the extremities, and a rubber-bulb syringe. Introducing the tube into the anus, and advancing it cautiously to the location of the tumor, he found a resistance, which was soon overcome by a stream of lukewarm water thrown against it; and then the tube, without much distress to the patient, was introduced sixty centimetres, when an examination of the abdomen showed that the tumor had entirely disappeared.

The remaining part of the day the patient slept at intervals, having no pain or other symptoms of illness, and at night the bowels discharged 1½ litres of offensive, bloody liquid, accompanied by flatus.

On the morning of the 3d of August, the patient experienced painful tenesmus, and discharged five or six scybala, with ½ litre of bloody fluid; the abdomen was slightly tender.

On the following day he had the first normal movement of the bowels, and subsequently convalescence progressed favorably.

